# Risk factors for complications after reduction mammaplasty: a systematic review and meta-analysis

**DOI:** 10.1186/s40001-025-02723-z

**Published:** 2025-06-02

**Authors:** Ran Ran, Hao Wang, Xing He, Junjie Li, Miao Yu, Exian Mou, Caiyang Liu

**Affiliations:** 1 Breast Surgery Center of Sichuan Cancer Hospital, Chengdu, 610041 China; 2 Department of Blood Transfusion, Jinniu District People’s Hospital, Chengdu, 610000 China; 3 Department of Cardiothoracic Surgery, The First People’s Hospital of Neijiang, No. 1866, West Section of Hanan Avenue, Shizhong District, Neijiang, 641000 Sichuan China

**Keywords:** Reduction mammaplasty, Postoperative complications, Risk factors, Meta-analysis

## Abstract

**Background:**

Reduction mammaplasty (RM) is a commonly performed plastic surgery procedure, but various postoperative complications have been noted. This study aims to identify and quantify the association between risk factors and the occurrence of postoperative complications following RM.

**Methods:**

We systematically reviewed PubMed, Medline, Cochrane Library and Web of Science for relevant publications, extracting suspected risk factors and associated postoperative complications. Meta-analyses were then conducted to evaluate their associations.

**Results:**

We included 61 articles encompassing 71,149 patients. Seven suspected risk factors and eighteen complications were identified. Five risk factors were significantly associated with any complications after RM: body mass index (BMI) ≥ 30 kg/m^2^ (OR 1.59, 95% CI 1.45–1.74, p = 0.000, I^2^ = 35.2%); smoking (OR 1.80, 95% CI 1.29–2.50, p = 0.000, I^2^ = 87.5%); diabetes (OR 1.41, 95% CI 1.11–1.79, p = 0.005, I^2^ = 11.2%); previous radiation therapy (OR 3.24, 95% CI 1.94–5.40, p = 0.000, I^2^ = 12.6%); and surgical techniques including inferior pedicle (IP) vs. superomedial pedicle (SMP) (OR 1.59, 95% CI 1.27–1.99, p = 0.000, I^2^ = 27.5%); IP vs. medial pedicle (MP) (OR 2.34, 95% CI 1.48–3.72, p = 0.000, I^2^ = 47.0%); and superior pedicle vs. SMP (OR 0.59, 95% CI 0.37–0.95, p = 0.028, I^2^ = 0.0%). Furthermore, BMI ≥ 30 kg/m^2^ was linked to higher risks of delayed healing, fat necrosis, wound infection, and dehiscence. Previous radiation therapy increased the risks of fat necrosis, wound infection, and seroma. Smoking was associated with higher risks of wound infection and dehiscence. Compared to MP, IP had a higher risk of wound dehiscence; compared to SMP, IP had a higher risk of wound infection but a lower risk of seroma (all P < 0.05).

**Conclusions:**

These findings highlight the importance of comprehensive preoperative risk assessment and individualized surgical planning to minimize postoperative complications and improve patient outcomes.

**Supplementary Information:**

The online version contains supplementary material available at 10.1186/s40001-025-02723-z.

## Background

Reduction mammaplasty (RM) is frequently performed to alleviate symptomatic macromastia or achieve contralateral breast symmetry following breast cancer surgery, aiming to enhance appearance and quality of life. RM, first performed by Paulus Aegineta between 625 and 690 for the treatment of gynecomastia, has gained popularity over the years [1, 2]. Although RM effectively alleviates physical and psychological issues associated with symptomatic macromastia and postoperative asymmetry, complications can occur at rates as high as 40%−50% [3–5].

Common postoperative complications include abnormal bleeding, wound issues, nipple-areola complex necrosis (NAC), breast asymmetry, loss of sensation in the nipple and areola, hematoma, and hypertrophic scarring. Rare complications, such as back pain, deep vein thrombosis, urinary tract infection, unplanned intubation, myocardial infarction, and sepsis, have also been documented [6–8]. Although characteristics such as obesity, smoking, diabetes, and age may increase susceptibility to postoperative complications, existing literature provides controversial results due to small sample sizes or other research limitations [3, 5, 9–11].

Considering the ongoing controversies in the existing literature and the limited range of risk factors and postoperative complications examined in previous meta-analyses, we conducted this study to identify and quantify the associations between various risk factors and postoperative complications following RM. Our findings aim to provide valuable guidance for developing individualized surgical strategies, minimizing postoperative complications, and ultimately improving patient outcomes.

## Methods

Our study was performed based on the guidelines of the Preferred Reporting Items for Systematic Reviews and Meta Analyses Statement [12].

### Criteria for inclusion and exclusion

Eligible articles met the following criteria: (i) patients must have undergone RM; (ii) studies must investigate the association between risk factors and complications post-RM; and (iii) articles must be full-text. Criteria for exclusion: (i) if other breast surgical procedures were mixed simultaneously; (ii) if the study was not a human-related case–control, cohort, cross sectional, or randomized controlled study; (iii) if there was a lack of essential data to calculate the odds ratios (OR) and 95% confidence intervals (CI); and (iv) if the articles were just case reports or letters to editors.

### Search strategy

We searched publications about RM and risk factors of postoperative complications on PubMed, MEDLINE, Cochrane Library and Web of Science without language restrictions before April 25, 2025, using the following key words: reduction mammaplasty, reduction mammoplasty, breast reduction, macromastia, gigantomastia, complication, postoperative complication. The search format was tailored to the syntax of each database. Additionally, we used Web of Science to retrieve key articles and identify further relevant citations. We also reviewed the references of the selected studies to find any other relevant papers. Two investigators independently screened titles and abstracts to exclude studies that did not meet the eligibility criteria. Next, the full texts of the potentially relevant studies were examined to finalize the selection of articles. Any disagreements were resolved through discussion; if consensus could not be reached, a senior reviewer made the final decision.

### Data extraction and quality assessment

Two investigators independently extracted raw data and assessed the quality of included studies using the Newcastle–Ottawa Scale (NOS). Any disagreements were resolved through discussion; if consensus could not be reached, a senior reviewer made the final decision. The NOS rating system evaluated studies based on three criteria: selection, comparability, and exposure. Respectively, these criteria included four, one, and three parameters. Scores ranged from 0 to 9, with studies considered high quality if they scored ≥ 7. For each eligible article, the following data were extracted: PMID, first author, publication year, study design, study duration, country or region, sample size, follow-up time, patient and treatment characteristics (including average age, average body mass index (BMI), proportion of smokers, average tissue resection weight per breast (TRW), proportion of patients with diabetes, proportion of patients with previous radiation therapy (PRT), and surgical techniques), and number of relevant complications. In view of sufficient data for meta-analysis, age, BMI, smoking, diabetes, TRW, surgical techniques (including inferior pedicle (IP), medial pedicle (MP), superior pedicle (SP), and superomedial pedicle (SMP)), and PRT were identified as suspected risk factors.

### Statistical analysis

All statistical analyses were performed using STATA version 12.0 (StataCorp LP, College Station, TX, USA). Meta-analysis results were illustrated with a forest plot, showing crude odds ratios (ORs) and 95% confidence intervals (CIs) to demonstrate the association between risk factors and complications. Subgroup analyses were conducted using available data on risk factors and specific complications. For age, BMI, and TRW, subgrouping was conducted based on the following thresholds: age ≥ 50 years, BMI ≥ 30, and TRW ≥ 1000. A p-value of < 0.05 was considered statistically significant. Cochran’s Q test and the I^2^ test were used to assess heterogeneity across the studies. p < 0.1 for Cochran’s Q test and I^2^ ≥ 50% indicated significant heterogeneity; therefore, random-effects models were used; otherwise, fixed-effects models were applied [13, 14]. Sensitivity analysis was conducted for meta-analyses including more than five publications to evaluate the impact of excluding each study individually on the overall results. Funnel plots were used to assess publication bias. Egger’s test were also performed for meta-analyses with more than five publications, where p > 0.05 indicated no publication bias [15].

## Results

### Study selection

**Fig. 1:**
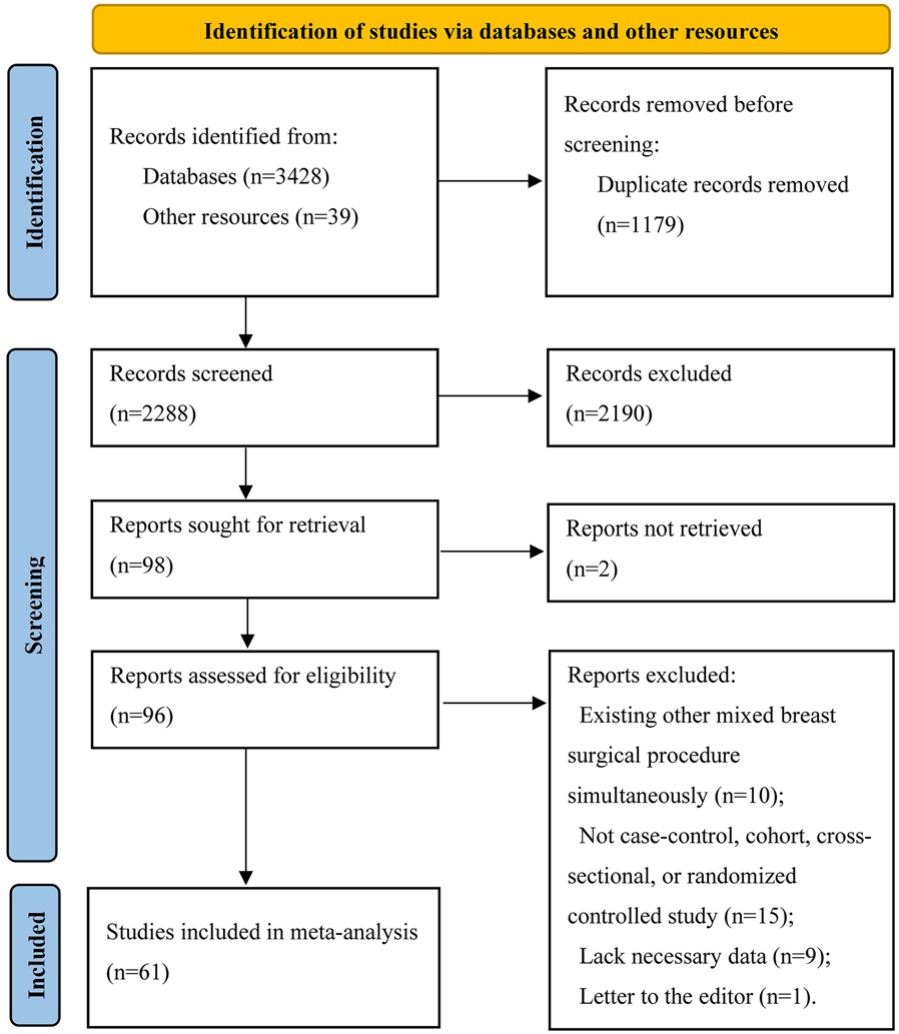
Flow diagram of search strategy and study selection

As shown in Fig. 1, a total of 3467 records were identified. After screening titles and abstracts, 1179 duplicate records and 2190 ineligible records were excluded. Two publications could not be retrieved. The full texts of 96 publications were assessed for eligibility. Ten publications were excluded because they involved concurrent breast surgical procedures. Fifteen publications were excluded for not being case–control, cohort, randomized controlled, or cross-sectional studies. Nine publications were excluded due to insufficient data and one was excluded as a letter to the editor. A list of excluded articles and the reasons for their exclusion are shown in Supplementary Table 1. Ultimately, sixty-one publications [3–5, 8, 10, 11, 16–70] were included in our study. Meta-analyses were conducted based on seven studies for age, nineteen studies for BMI, seven studies for TRW, six studies for diabetes, five studies for PRT, and nineteen studies for smoking. Three studies compared IP and MP, twelve studies compared IP and SMP, six studies compared IP and SP, and three studies compared SP and SMP.

### Characteristics of study and patient

The characteristics of the sixty-one publications are presented in Table 1. According to the NOS, the quality scores of the included studies ranged from six to nine. These publications, spanning from 1992 to 2024, provide evidence from six prospective studies, fifty-two retrospective studies, and three randomized controlled trials. A total of 71,149 patients were included in the sixty-one studies. Based on the reported data, the mean age of patients was 43.3 ± 8.9 years, the mean BMI was 30.7 ± 2.3 kg/m^2^, and the mean TRW was 848.5 ± 410.0 g. Across the reported studies, an average of 16.5% of patients were smokers, 4.5% had diabetes, and 24.0% had PRT. The most frequently used surgical techniques were IP, SMP, and SP, while MP and free nipple grafting were reported in five and four studies, respectively. Occasionally, other surgical techniques such as modified Robertson wide pattern, modified Lejour technique, vertical bi-pedicle, and central mound technique were employed. Based on the available data, the incidence of any complications ranged from 3.8% to 57.9%, with a mean of 12.3%. The incidences of the other seventeen specific complications are presented in Table 2.

**Table 1:** Study characteristics

References	NOS	Design	Years	Country	Sample size	Follow-up(month)	Age (year)	BMI (kg/m^2^)	Smokers (%)	TRW(g)	Diabetes (%)	Radiation therapy (%)	Techniques (%)
Hagen et al. [16]	8	R-COH	2001–2002	UK	71	0.3–15.3	39.0	27.5	33.8	648.0	NR	NR	NR
HEFTER et al. [17]	7	R-COH	1985–2000	Norway	101	NR	37.9	27.0	NR	688.0	NR	NR	NR
Chan et al. [18]	7	R-COH	2002–2004	UK	169	NR	36.2	26.7	38.5	713.8	NR	NR	IP:63.3; SP:29.0; VS:4.1; FNG:3.6
Dex et al. [19]	8	R-COH	1998–2003	UK	220	18.7	35.1	28.5	NR	789.0	NR	NR	VS:37.2; Inverted-T:54.5; FNG:8.2
Roehl et al. [20]	8	R-COH	1996–2006	USA	179	1.0–12.0	35.0	34.0	4.2	NR	NR	NR	IP:74.9; VS:4.5; FNG:20.6
Gust et al. [8]	8	R-COH	2006–2010	USA	2492	NR	42.2	31.7	12.0	NR	NR	NR	NR
Nelson et al. [3]	8	R-COH	2005–2011	USA	4545	1.0	NR	NR	11.7	NR	5.1	NR	NR
Nelson et al. [21]	8	R-COH	2005–2010	USA	3537	1.0	43.2	31.6	12.1	NR	NR	NR	NR
Karamanos et al. [10]	7	R-COH	2005–2010	USA	2779	NR	42.7	31.6	11.7	NR	NR	NR	NR
Srinivasaiah et al. [22]	NA	RCT	2001–2002	UK	67	NR	39.3	28.5	43.3	1386.7	NR	NR	NR
Hillam et al. [23]	7	R-COH	2009–2014	USA	13,503	NR	44.3	31.1	10.3	NR	NR	NR	NR
Baltodano et al. [24]	8	R-COH	2005–2012	USA	7068	1.0	43.7	NR	10.9	NR	NR	NR	NR
Simpson et al. [25]	8	R-COH	2006–2015	USA	16,812	1.0	43.3	31.2	10.1	NR	NR	NR	NR
Young et al. [26]	8	R-COH	2013–2015	USA	9110	1.0	42.1	29.9	9.4	NR	4.6	NR	NR
Davé et al. [27]	7	R-COH	2005–2017	USA	1854	NR	69.0	NR	3.7	NR	11.4	NR	NR
Fairchild et al. [28]	7	R-COH	2017–2020	USA	542	1.0	17.0	30.0	NR	NR	NR	NR	NR
Toplu et al. [11]	9	R-COH	2013–2018	Turkey	186	48.0	45.0	29.0	4.3	1100.0	5.9	NR	IP: 17.2; SMP: 53.2; SP: 29.6
Coady-Fariborzian et al. [29]	8	R-COH	2000–2020	USA	115	3.0	43.0	30.0	NR	1272.0	2.6	NR	IP: 45.2; SP: 54.8
Setela et al. [30]	9	R-COH	1998–2003	Finland	273	6.0	43.0	28.0	NR	730.5	NR	NR	IP: 64.1; SP: 34.4
Shah et al. [5]	7	R-COH	1999–2004	UK	306	3.0	36.8	27.6	NR	NR	NR	NR	NR
Antony et al. [31]	7	R-COH	2009–2012	USA	100	60.0	31.5	31.3	11.1	827.5	NR	NR	SMP: 50.0; IFP: 50.0
Palve et al. [32]	7	R-COH	2014–2020	Finland	760	32.4	51.0	27.8	6.1	460.0	4.6	NR	SMP: 63.2; IP: 26.5; SP: 10.3
Ogunleye et al. [33]	6	R-COH	2008–2015	USA	124	NR	39.2	30.5	3.2	692.1	NR	NR	SMP: 73.4; IP: 26.6
Cunning et al. [34]	7	R-COH	2017–2019	USA	101	3.0	38.0	29.6	NR	NR	NR	NR	SMP: 59.2; IP: 40.8
Ulusal et al. [35]	8	R-COH	2010–2016	Turkey	40	8.0–72.0	44.2	31.8	NR	1533.0	NR	NR	SP: 100
Bustos et al. [36]	8	R-COH	2014–2019	USA	288	3.9	43.7	31.5	32.6	699.6	NR	NR	IP: 100
O’Grady et al. [37]	9	R-COH	2000–2002	Canada	133	5.1	NR	NR	NR	NR	NR	NR	IP: 100
Cunningham et al. [4]	8	P-COH	1997–1998	USA	179	9.0	39.4	29.7	12.6	1662.6	NR	NR	IP: 77.5; SP: 20.3; MP: 1.1; Glandular: 1.1
Bikhchandani et al. [38]	7	R-COH	1999–2004	UK	402	NR	34.3	28.9	27.9	665.1	NR	NR	IP: 100
Chun et al. [39]	8	R-COH	1995–2007	USA	675	NR	37.5	31.0	8.9	1696.0	NR	NR	WP: 80.7; MR: 18.7; VS: 0.6
Ngaage et al. [40]	7	R-COH	2006–2013	USA	70	72.0	41.0	34.5	NR	NR	NR	NR	NR
Azzam et al. [41]	6	R-COH	1996–2002	German	115	NR	35.2	28.8	0.4	815.2	NR	NR	ML: 100
Parrett et al. [42]	8	R-COH	2004–2008	USA	12	10.0	57.0	29.9	0.0	452.5	NR	50.0	IP: 41.7; Others: 58.3
Dal Cin [43]	8	R-CC	1980–2007	Canada	9	32.9	56.2	30.0	NR	577.3	NR	50.0	IP: 88.9; SP: 11.1
Weichman [44]	8	R-COH	2008–2013	USA	13	NR	50.2	26.8	0.0	320.5	NR	50.0	CMT: 100
Ercan [45]	9	R-COH	2015–2023	Turkey	190	6.0	48.4	28.5	18.4	608.4	7.9	NR	IP: 36.3; SP: 63.7
Girard et al. [46]	8	R-COH	2011–2016	France	1306	6.0	38.9	27.3	22.9	530.7	7.9	NR	SP: 100
Adebagbo et al. [47]	8	R-COH	2017–2023	USA	178	NR	41.4	30.3	22.5	519.4	3.4	NR	IP: 71.9; SP: 28.1
Roje et al. [48]	8	R-COH	1999–2011	Croatia	59	6.0	47.0	NR	22.0	1057.0	NR	NR	VP: 72.9; SMP: 10.2; IP: 10.2; FNG: 6.8
Güemes et al. [49]	8	P-COH	NR	Spain	121	12.0	40.7	29.6	34.7	1785.5	NR	NR	IP: 100
Deliaert et al. [50]	7	P-COH	2006–2007	Netherlands	43	9.0	35.9	26.2	30.2	437.3	NR	NR	Mediocranial pedicle: 100
Sandsmark et al. [51]	6	R-COH	1984–1990	Norway	292	51.0	30.4	NR	NR	547.0	NR	NR	SMP: 82.3; IP: 9.4; Other: 8.3
Chang et al. [52]	7	R-COH	1987–1994	USA	172	16.0	33.0	31.6	NR	1946.0	NR	NR	IP: 100
Platt et al. [53]	NA	RCT	NR	UK	30	2.0	33.0	26.3	NR	635.0	NR	NR	IP: 100
Zoumaras et al. [54]	6	R-COH	2002–2005	Australia	191	6.0	39.8	28.8	NR	1006.8	NR	NR	IP: 49.7; MP: 50.3
Cruz-Korchin et al. [55]	NA	RCT	1999–2002	USA	208	6.0	30.0	26.5	NR	550.5	NR	NR	IP: 50.5; MP: 49.5
Kreithen et al. [56]	6	R-COH	1999–2002	USA	112	NR	33.3	32.2	20.5	997.3	NR	NR	IP: 62.5; SP: 37.5
Gamboa-Bobadilla et al. [57]	6	R-COH	2000–2003	USA	86	43.0	35.1	36.8	NR	1362.3	NR	NR	IP: 100
James et al. [58]	6	R-COH	1997–2005	USA	84	18.0	38.0	29.0	NR	560.3	NR	NR	IP: 58.3; MP: 41.7
Eleanor et al. [59]	8	R-COH	2007–2014	USA	236	6.0	38.3	NR	25.0	823.0	3.4	NR	SMP: 100
Webb et al. [60]	7	R-COH	1997–2008	USA	67	3.0	17.1	27.9	NR	1358.3	NR	NR	IP: 70.1
Gulcelik et al. [61]	7	P-CC	NR	Turkey	153	12.0	48.8	29.0	17.6	958.0	NR	NR	LPF: 81.0; UPF: 19.0
Braig et al. [62]	7	R-COH	1998–2011	German	50	12.7	44.4	28.6	12.0	842.0	NR	NR	SMP: 100
Egro et al. [63]	7	R-COH	2005–2012	USA	160	47.4	53.8	33.0	12.5	616.0	8.8	15.6	IP: 19.4; SP: 71.3
Mendonc et al. [64]	8	R-COH	1999–2009	Brazil	144	47.0	49.4	25.9	19.4	NR	6.3	26.4	SMP: 41.7; SP: 29.2; IP: 22.2; SLP:7.0
Patel et al. [65]	6	R-COH	2003–2009	USA	16	34.0	52.5	31.5	18.8	338.2	25.0	31.3	SMP: 9.4; MP: 31.3; IP: 28.1; CMT:31.3
Kemaloğlu et al. [66]	7	P-COH	NR	Turkey	50	19.6	40.2	31.9	4.0	1341.3	NR	NR	IP: 50.0; SMP: 50.0
Kulkarni et al. [67]	8	R-COH	2011–2017	USA	60	6.3	16.7	28.4	3.3	520.5	NR	NR	IP: 80.0; SMP: 20.0
Yeğin et al. [68]	7	R-COH	2017–2020	Turkey	93	NR	35.8	33.5	54.8	NR	NR	NR	IP: 28.0; SMP: 72.0
Sapino et al. [69]	9	R-COH	2015–2017	Switzerland	58	24.0	34.0	28.8	19.0	662.2	NR	NR	IP: 37.9; SMP: 62.1
Özçelik [70]	8	P-COH	2010–2022	Tu¨rkiye	40	6.0	37.1	27.1	NR	643.3	NR	NR	SP: 50.0; SMP: 50.0

R-COH: retrospective cohort; P-COH: prospective cohort; NOS: Newcastle–Ottawa Scale; RCT: randomized controlled trial; P-CC: prospective case control; R-CC: retrospective case control; TRW: tissue resection weight per breast; BMI: body mass index; NR: not reported; NA: not applicable; IP: inferior pedicle; SP: superior pedicle; SMP: superomedial pedicle; VS: verticle scar; FNG: free nipple graft; MP: medial pedicle; WP: wise pattern; MR: modified robertson; ML: modified Lejour technique; CMT: central mound technique; VP: Vertical bi-pedicle; LPF: lower pediculated flap; UPL: upper pediculated flap; SLP: superior lateral pedicle

**Table 2:** Incidence of relevant complications after reduction mammoplasty

References	AC (%)	WI (%)	WD (%)	MC (%)	HT (%)	NAC (%)	FN (%)	SM (%)	RP (%)	30-D-R (%)	SN (%)	MSB (%)	NSL (%)	TN (%)	DI (%)	DH (%)	SR (%)	HS (%)
Hagen et al. [16]	11 (15.5)	NR	NR	NR	NR	NR	NR	NR	NR	NR	NR	NR	NR	NR	NR	NR	NR	NR
HEFTER et al. [17]	10 (9.9)	NR	NR	NR	NR	NR	NR	NR	NR	NR	NR	NR	NR	NR	NR	NR	NR	NR
Chan et al. [18]	71 (42.1)	29 (17.2)	27 (16.0)	NR	NR	NR	6 (3.6)	NR	9 (5.3)	NR	NR	NR	NR	NR	NR	NR	NR	NR
Dex et al. [19]	31 (14.1)	NR	26 (11.8)	NR	NR	1 (0.5)	4 (1.8)	NR	NR	NR	NR	NR	NR	NR	NR	NR	NR	NR
Roehl et al. [20]	90 (50.3)	NR	NR	NR	NR	NR	NR	NR	NR	NR	NR	NR	NR	NR	NR	NR	NR	NR
Gust et al. [8]	112 (4.5)	78 (3.1)	23 (0.9)	14 (0.6)	NR	3 0.1)	NR	NR	NR	NR	NR	NR	NR	NR	NR	NR	NR	NR
Nelson et al. [3]	275 (6.1)	148 (3.3)	37 (0.8)	43 (0.9)	NR	NR	NR	NR	82 (1.8)	NR	NR	NR	NR	NR	NR	NR	NR	NR
Nelson et al. [21]	206 (5.8)	NR	NR	NR	NR	NR	NR	NR	NR	NR	NR	NR	NR	NR	NR	NR	NR	NR
Karamanos et al. [10]	159 (5.7)	84 (3.0)	25 (0.9)	NR	6 (0.2)	NR	NR	NR	54 (1.9)	NR	NR	NR	NR	NR	NR	NR	NR	NR
Srinivasaiah et al. [22]	16 (23.9)	9 (13.4)	2 (3.0)	NR	1 (1.5)	NR	3 (4.5)	NR	NR	NR	NR	NR	NR	NR	NR	NR	NR	NR
Hillam et al. [23]	587 (4.2)	428 (3.2)	96 (0.7)	70 (0.5)	43 (0.4)	NR	NR	NR	NR	NR	NR	NR	NR	NR	NR	NR	NR	NR
Baltodano et al. [24]	268 (3.8)	218 (3.1)	50 (0.7)	NR	NR	NR	NR	NR	NR	NR	NR	NR	NR	NR	NR	NR	NR	NR
Simpson et al. [25]	3535 (21.0)	NR	NR	NR	NR	NR	NR	NR	NR	NR	NR	NR	NR	NR	NR	NR	NR	NR
Young et al. [26]	641 (7.0)	290 (3.2)	61 (0.7)	290 (3.2)	NR	NR	NR	NR	NR	NR	NR	NR	NR	NR	NR	NR	NR	NR
Davé et al. [27]	114 (6.1)	NR	NR	NR	NR	NR	NR	NR	NR	NR	NR	NR	NR	NR	NR	NR	NR	NR
Fairchild et al. [28]	25 (4.6)	13 (2.4)	4 (0.7)	NR	NR	NR	NR	NR	NR	10 (1.8)	NR	NR	NR	NR	NR	NR	NR	NR
Toplu et al. [11]	13 (6.9)	1 (0.53)	2 (1.1)	NR	3 (1.6)	3 (1.6)	2 (1.1)	NR	6 (3.2)	NR	NR	NR	1 (0.53)	NR	NR	NR	NR	1 (0.53)
Coady-Fariborzian et al. [29]	48 (41.7)	NR	NR	NR	NR	NR	NR	NR	NR	NR	NR	NR	NR	NR	NR	NR	NR	NR
Setela et al. [30]	143 (52.4)	95 (34.8)	NR	NR	NR	7 (2.6)	8 (2.9)	NR	NR	NR	49 (17.9)	NR	NR	NR	NR	NR	NR	NR
Shah et al. [5]	165 (53.9)	43 (14.1)	81 (26.5)	NR	52 (17.0)	NR	NR	NR	NR	NR	NR	NR	NR	NR	NR	NR	NR	NR
Antony et al. [31]	56 (56.0)	3 (3.0)	NR	NR	1 (1.0)	1 (1.0)	NR	7 (7.0)	2 (2.0)	NR	NR	18 (18.0)	24 (24.0)	NR	NR	NR	NR	NR
Palve et al. [32]	290 (38.2)	242 (31.8)	NR	NR	24 (3.2)	NR	NR	27 (3.6)	NR	NR	NR	NR	NR	4 (0.5)	3 (0.4)	NR	NR	NR
Ogunleye et al. [33]	39 (31.5)	11 (8.9)	11 (8.9)	NR	10 (8.1)	NR	7 (5.6)	5 (4.0)	NR	NR	2 (1.6)	NR	NR	NR	NR	NR	NR	NR
Cunning et al. [34]	18 (17.8)	2 (2.0)	3 (3.0)	NR	2 (2.0)	1 (1.0)	2 (2.0)	NR	NR	NR	NR	NR	NR	NR	NR	10 (10.0)	NR	NR
Ulusal et al. [35]	12 (30.0)	NR	NR	NR	NR	NR	NR	NR	NR	NR	NR	NR	NR	NR	NR	NR	NR	NR
Bustos et al. [36]	48 (16.7)	NR	NR	NR	NR	NR	NR	NR	NR	NR	NR	NR	NR	NR	NR	NR	NR	NR
O’Grady et al. [37]	149 (56.0)	NR	NR	NR	NR	NR	NR	NR	NR	NR	NR	NR	NR	NR	NR	NR	NR	NR
Cunningham et al. [4]	77 (43.0)	NR	NR	NR	NR	NR	NR	NR	NR	NR	NR	NR	NR	NR	NR	NR	NR	NR
Bikhchandani et al. [38]	94 (23.4)	NR	NR	NR	NR	NR	NR	NR	NR	NR	NR	NR	NR	NR	NR	NR	NR	NR
Chun et al. [39]	75 (11.1)	NR	NR	NR	NR	NR	NR	NR	NR	NR	NR	NR	NR	NR	NR	NR	NR	NR
Ngaage et al. [40]	17 (24.3)	NR	NR	NR	NR	NR	NR	NR	NR	NR	NR	NR	NR	NR	NR	NR	NR	NR
Azzam et al. [41]	93 (40.4)	8 (3.5)	49 (21.3)	NR	10 (4.3)	NR	NR	13 (5.7)	NR	NR	NR	NR	NR	NR	NR	NR	35 (15.2)	NR
Parrett et al. [42]	5 (41.7)	3 (25.0)	1 (8.3)	NR	NR	0 (0.0)	2 (16.7)	5 (41.7)	NR	NR	NR	NR	NR	NR	NR	NR	NR	NR
Dal Cin [43]	NR	6 (66.7)	NR	NR	NR	1 (11.1)	0 (0.0)	NR	NR	NR	NR	NR	NR	NR	NR	NR	3 (33.3)	3 (33.3)
Weichman [44]	NR	2 (15.4)	NR	NR	1 (7.7)	0 (0.0)	2 (7.7)	NR	NR	NR	NR	NR	NR	NR	NR	NR	NR	4 (30.8)
Ercan [45]	58 (30.5)	19 (10.0)	24 (12.6)	0 (0.0)	4 (2.1)	1 (0.5)	10 (5.3)	NR	NR	NR	NR	NR	NR	NR	NR	NR	NR	NR
Girard et al. [46]	284 (21.8)	6 (0.5)	221 (16.9)	0 (0.0)	32 (2.5)	32 (2.5)	176 (13.5)	NR	0 (0.0)	NR	NR	NR	NR	NR	NR	NR	NR	NR
Adebagbo et al. [47]	71 (39.9)	28 (15.7)	9 (5.1)	NR	1 (0.6)	NR	NR	5 (2.8)	13 (7.3)	1 (0.6)	NR	NR	14 (8.0)	NR	NR	NR	NR	6 (3.4)
Roje et al. [48]	33 (55.9)	4 (6.7)	NR	3 (5.1)	3 (5.1)	2 (3.4)	1 (1.7)	9 (15.3)	NR	NR	NR	NR	4 (6.7)	NR	NR	8 (13.6)	NR	3 (5.1)
Güemes et al. [49]	70 (57.9)	2 (1.7)	NR	NR	4 (3.3)	2 (1.7)	3 (2.5)	NR	NR	NR	1 (0.8)	NR	NR	NR	NR	14 (11.6)	NR	19 (15.7)
Deliaert et al. [50]	NR	NR	20 (46.5)	NR	NR	NR	NR	NR	NR	NR	NR	NR	NR	NR	NR	NR	NR	NR
Sandsmark et al. [51]	94 (32.9)	8 (2.9)	20 (46.5)	NR	40 (14.4)	4 (1.4)	11 (4.0)	NR	NR	NR	NR	NR	18 (6.3)	NR	NR	NR	13 (4.7)	NR
Chang et al. [52]	72 (41.9)	12 (7.0)	5 (2.9)	NR	4 (2.4)	2 (1.2)	2 (1.2)	NR	NR	NR	NR	NR	7 (4.1)	NR	NR	NR	NR	18 (10.5)
Platt et al. [53]	16 (53.3)	NR	13 (43.3)	NR	NR	NR	NR	NR	NR	NR	NR	NR	NR	NR	NR	NR	NR	NR
Zoumaras et al. [54]	102 (53.4)	24 (12.6)	48 (25.1)	NR	11 (5.8)	1 (0.5)	10 (5.2)	0 (0)	NR	NR	NR	NR	NR	NR	NR	NR	NR	NR
Cruz-Korchin et al. [55]	16 (7.7)	2 (1.0)	7 (3.4)	NR	2 (1.0)	2 (1.0)	NR	3 (1.4)	NR	NR	NR	NR	NR	NR	NR	NR	NR	NR
Kreithen et al. [56]	82 (73.2)	NR	NR	NR	4 (3.6)	7 (6.2)	NR	NR	NR	NR	NR	NR	NR	NR	NR	NR	NR	NR
Gamboa-Bobadilla et al. [57]	NR	16 (18.6)	NR	NR	NR	NR	7 (8.1)	NR	NR	NR	NR	NR	NR	NR	NR	24 (27.9)	NR	NR
James et al. [58]	19 (22.6)	NR	NR	NR	4 (4.8)	NR	6 (7.1)	3 (3.6)	4 (4.8)	NR	NR	NR	NR	NR	NR	15 (17.9)	NR	13 (15.5)
Eleanor et al. [59]	67 (28.4)	12 (5.1)	NR	NR	8 (3.4)	0 (0.0)	18 (7.6)	5 (2.1)	NR	NR	NR	NR	NR	NR	NR	27 (11.4)	NR	NR
Webb et al. [60]	34 (50.7)	2 (3.0)	19 (28.4)	NR	−1.5	NR	NR	NR	NR	NR	NR	NR	NR	NR	NR	NR	NR	5 (7.5)
Gulcelik et al. [61]	28 (18.3)	4 (2.6)	6 (4.0)	NR	3 (1.9)	1 (0.6)	NR	8 (5.2)	NR	NR	NR	NR	NR	NR	NR	6 (4%)	NR	NR
Braig et al. [62]	21 (42.0)	6 (12.0)	3 (6.0)	NR	3 (6.0)	1 (2.0)	NR	NR	18 (36.0)	NR	NR	NR	NR	NR	NR	NR	NR	NR
Egro et al. [63]	45 (28.1)	8 (5.0)	5 (3.1)	NR	4 (2.5)	1 (0.6)	3 (1.9)	3 (1.9)	NR	NR	2 (1.3)	NR	NR	NR	NR	13 (8.1)	NR	7 (4.4)
Mendonc et al. [64]	44 (30.1)	6 (4.2)	8 (5.6)	NR	1 (0.7)	4 (2.8)	10 (6.9)	NR	17 (11.8)	NR	15 (10.4)	NR	NR	NR	NR	NR	NR	NR
Patel et al. [65]	7 (43.8)	NR	NR	NR	NR	NR	NR	NR	NR	NR	NR	NR	NR	NR	NR	NR	NR	NR
Kemaloğlu et al. [66]	6 (12.0)	NR	3 (6.0)	NR	NR	1 (2.0)	NR	NR	NR	NR	NR	NR	2 (4.0)	NR	NR	NR	NR	NR
Kulkarni et al. [67]	14 (23.3)	1 (1.7)	6 (10.0)	NR	0 (0.0)	1 (1.7)	2 (3.3)	0 (0.0)	1 (1.7)	NR	0 (0.0)	NR	0 (0.0)	NR	NR	7 (11.7)	NR	1 (1.7)
Yeğin et al. [68]	10 (10.8)	NR	NR	NR	NR	2 (2.2)	NR	NR	NR	NR	NR	NR	NR	NR	NR	NR	NR	NR
Sapino et al. [69]	30 (51.7)	3 (5.2)	2 (3.4)	NR	NR	0 (0.0)	NR	2 (3.4)	NR	NR	NR	NR	NR	NR	NR	NR	NR	13 (22.8)
Özçelik [70]	15 (30.0)	NR	1 (2.0)	NR	1 (2.0)	NR	NR	2 (4.0)	NR	NR	NR	NR	NR	NR	NR	2 (4.0)	NR	NR

NR: not reported; AC: any complications; WI: wound infection; WD: wound dehiscence; MC: medical complications; HT: haematoma; NAC: nipple-areola complex necrosis; FN: fat necrosis; SM: seroma; RP: reoperation; 30-D-R: 30-day-readmission; SN: skin necrosis; MSB: minor skin breakdown; NSL: nipple sensory loss; TN: tissue necrosis; DI: deep infection; DH: delayed healing; SR: scar revision; HS: hypertrophic scarring

### Association between risk factors and complications

A total of fifty-nine associations were evaluated. A summary of meta-analysis results on suspected risk factors and complications is shown in Table 3.

**Table 3:** Summary of meta-analysis results on suspected risk factors and complications

Suspected risk factors	Complications	No. of study	OR	95% CI	P	I^2^ (%)	P_H_	P (Egger)
Age	Any complications	7	0.99	0.64–1.52	0.961	74.8	0.001	0.200
BMI	Any complications	19	**1.59**	**1.45–1.74**	**0.000**	35.2	0.066	0.503
	Delayed healing	3	**1.93**	**1.03–3.63**	**0.041**	0.0	0.683	–
	Fat necrosis	3	**2.56**	**1.01–6.51**	**0.048**	0.0	0.607	–
	Hematoma	3	0.64	0.22–1.84	0.410	34.9	0.215	–
	Hypertrophic scarring	2	1.46	0.61–3.50	0.397	14.2	0.280	–
	Medical complications	2	1.56	0.90–2.69	0.111	0.0	0.423	–
	Nipple-areola complex necrosis	2	0.24	0.01–5.11	0.361	–	–	–
	Wound infection	10	**1.54**	**1.29–1.84**	**0.000**	0.0	0.924	0.044
	Wound dehiscence	8	**2.19**	**1.27–3.78**	**0.005**	60.8	0.013	0.161
Diabetes	Any complications	6	**1.41**	**1.11–1.79**	**0.005**	11.2	0.344	0.957
Radiation	Any complications	5	**3.24**	**1.94–5.40**	**0.000**	12.6	0.334	0.423
	Delayed healing	2	1.50	0.42–5.30	0.532	11.0	0.289	–
	Fat necrosis	4	**2.74**	**1.03–7.33**	**0.045**	0.0	0.526	–
	Hematoma	3	0.53	0.09–3.29	0.499	0.0	0.899	–
	Hypertrophic scarring	3	1.60	0.52–4.96	0.416	42.8	0.174	–
	Wound infection	4	**4.62**	**1.57–13.63**	**0.005**	13.0	0.328	–
	Nipple-areola complex necrosis	5	2.52	0.60–10.59	0.207	0.7	0.365	0.544
	Seroma	2	**7.65**	**1.39–41.93**	**0.019**	3.4	0.309	–
	Wound dehiscence	3	2.45	0.81–7.40	0.113	0.0	0.817	–
Smoking	Any complications	19	**1.80**	**1.29–2.50**	**0.000**	87.5	0.000	0.148
	Hematoma	5	0.66	0.29–1.49	0.319	16.6	0.309	0.843
	Medical complications	2	0.61	0.15–2.47	0.488	74.2	0.049	–
	Wound dehiscence	8	**1.84**	**1.35–2.52**	**0.000**	36.5	0.138	0.606
	Wound infection	7	**1.86**	**1.57–2.22**	**0.000**	0.0	0.516	0.365
TRW	Any complications	7	1.64	0.99–2.71	0.053	63.2	0.012	0.983
Surgical technique (IP VS MP)	Any complications	3	**2.34**	**1.48–3.72**	**0.000**	47.0	0.152	–
	Fat necrosis	2	0.88	0.32–2.44	0.805	0.0	0.473	–
	Hematoma	3	0.37	0.13–1.07	0.067	0.0	0.719	–
	Nipple-areola complex necrosis	2	0.59	0.08–4.55	0.616	0.0	0.618	–
	Seroma	3	0.41	0.07–2.28	0.309	0.0	0.844	–
	Wound dehiscence	2	**3.39**	**1.83–6.29**	**0.000**	43.5	0.183	–
	Wound infection	2	2.06	0.88–4.85	0.096	0.0	0.583	–
Surgical technique (IP VS SMP)	Any complications	12	**1.59**	**1.27–1.99**	**0.000**	27.5	0.175	0.979
	Fat necrosis	5	1.48	0.55–3.95	0.435	0.0	0.953	0.470
	Hematoma	6	1.05	0.59–1.85	0.879	36.1	0.166	0.432
	Wound infection	8	**2.15**	**1.57–2.94**	**0.000**	7.8	0.370	0.789
	Nipple-areola complex necrosis	10	1.50	0.62–3.66	0.369	0.0	0.902	0.886
	Nipple sensory loss	3	0.70	0.19–2.62	0.593	42.6	0.175	–
	Seroma	4	**0.28**	**0.09–0.80**	**0.018**	0.0	0.430	–
	Skin necrosis	2	0.41	0.05–3.33	0.407	0.0	0.864	–
	Wound dehiscence	5	1.55	0.67–3.57	0.304	45.3	0.120	0.558
	Hypertrophic scarring	3	1.37	0.46–4.12	0.575	0.0	0.913	–
	Reoperation	3	0.53	0.16–1.77	0.298	1.0	0.364	–
Surgical technique (IP VS SP)	Any complications	6	1.32	0.74–2.36	0.346	75.1	0.001	0.944
	Nipple sensory loss	2	0.91	0.32–2.64	0.865	27.9	0.239	–
	Fat necrosis	3	0.79	0.32–1.99	0.617	0.0	0.695	–
	Hematoma	4	0.95	0.28–3.28	0.936	0.0	0.892	–
	Hypertrophic scarring	2	5.36	0.30–96.92	0.256	–	–	–
	Wound infection	5	1.50	0.61–3.69	0.377	84.4	0.000	0.775
	Nipple-areola complex necrosis	4	0.38	0.14–1.00	0.050	0.0	0.823	–
	Reoperation	2	0.41	0.14–1.19	0.100	0.0	0.878	–
	Seroma	2	0.99	0.19–5.19	0.989	0.0	0.451	–
	Wound dehiscence	3	0.94	0.45–1.96	0.873	0.0	0.546	–
Surgical technique (SP VS SMP)	Any complications	3	**0.59**	**0.37–0.95**	**0.028**	0.0	0.417	–
	Hematoma	3	0.43	0.11–1.65	0.220	19.7	0.288	–
	Wound infection	2	0.68	0.39–1.19	0.179	0.0	0.931	–
	Nipple-areola complex necrosis	2	9.30	0.44–197.24	0.152	–	–	–
	Wound dehiscence	2	0.43	0.04–4.27	0.473	0.0	0.790	–

BMI: body mass index; IP: inferior pedicle; MP: medial pedicle; SMP: superomedial pedicle; SP: superior pedicle; TRW: tissue resection weight per breast

Bold values indicate statistically significant associations (p＜0.05 or as defined by the 95% confidence interval excluding the null value) in the meta-analysis

#### Associations of age, diabetes, and TRW with complications

Meta-analyses were conducted using the available data to evaluate risk factors based on ORs, 95% CIs, and p-values. Pooled results from seven studies showed that age ≥ 50 years was not significantly associated with risk of any complications (OR = 0.99, 95% CI 0.64–1.52, p = 0.961; I^2^ = 74.8%). A meta-analysis of six studies demonstrated a significantly higher incidence of any complications in diabetic patients following RM (OR = 1.41, 95% CI 1.11–1.79, p = 0.005; I^2^ = 11.2%). Another meta-analysis including seven studies found no significant association between TRW ≥ 1000 g and any complications (OR = 1.64, 95% CI 0.99–2.71, p = 0.053; I^2^ = 63.2%). However, there were insufficient data to support subgroup analyses for these three risk factors.

#### Associations of BMI with complications

**Fig. 2:**
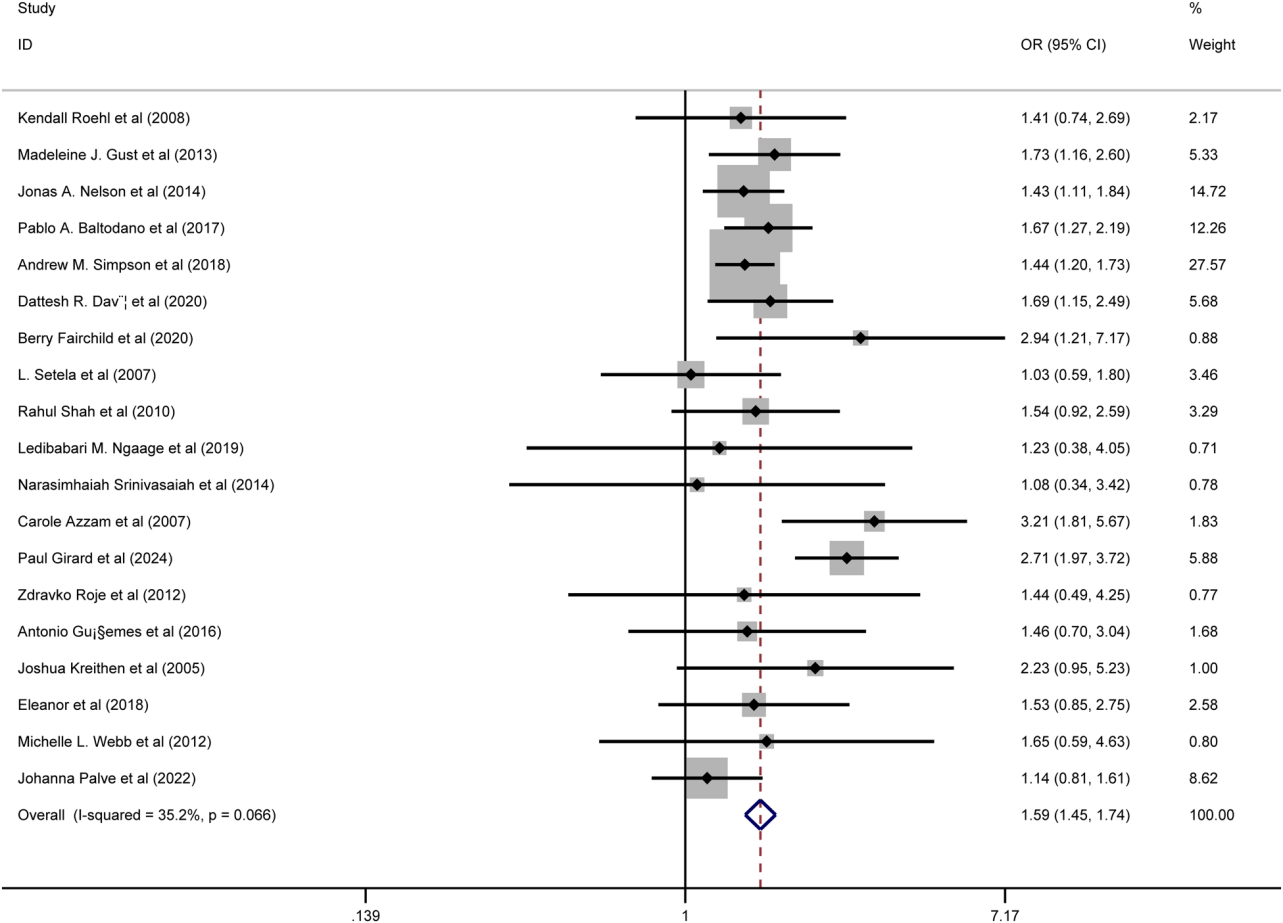
Association between body mass index ≥ 30 kg/m^2^ and any complications

Pooled results from nineteen studies showed that BMI ≥ 30 kg/m^2^ was significantly associated with an increased risk of any complications (OR = 1.59, 95% CI 1.45–1.74, p = 0.000; I^2^ = 35.2%) Fig. 2. Subgroup analyses demonstrated a significantly higher incidence of delayed healing (OR = 1.93, 95% CI 1.03–3.63, p = 0.041; I^2^ = 0.0%), fat necrosis (OR = 2.56, 95% CI 1.01–6.56, p = 0.048; I^2^ = 0.0%), wound infection (OR = 1.54, 95% CI 1.29–1.84, p = 0.000; I^2^ = 0.0%), and wound dehiscence (OR = 2.19, 95% CI 1.27–3.78, p = 0.005; I^2^ = 60.8%) in patients with BMI ≥ 30 kg/m^2^ following RM. No significant associations were found between BMI and hematoma, hypertrophic scarring, medical complications, or NAC.

#### Associations of PRT with complications

Pooled results from five studies showed that PRT was significantly associated with an increased risk of any complications (OR = 3.24, 95% CI 1.94–5.40, p = 0.000; I^2^ = 12.6%). Subgroup analyses demonstrated a significantly higher incidence of fat necrosis (OR = 2.74, 95% CI 1.03–7.33, p = 0.045; I^2^ = 0.0%), wound infection (OR = 4.62, 95% CI 1.57–13.63, p = 0.005; I^2^ = 13.0%), and seroma (OR = 7.65, 95% CI 1.39–41.93, p = 0.019; I^2^ = 3.4%) in patients with PRT following RM. No significant associations were found between PRT and delayed healing, hematoma, hypertrophic scarring, or NAC.

#### Associations of smoking with complications

**Fig. 3:**
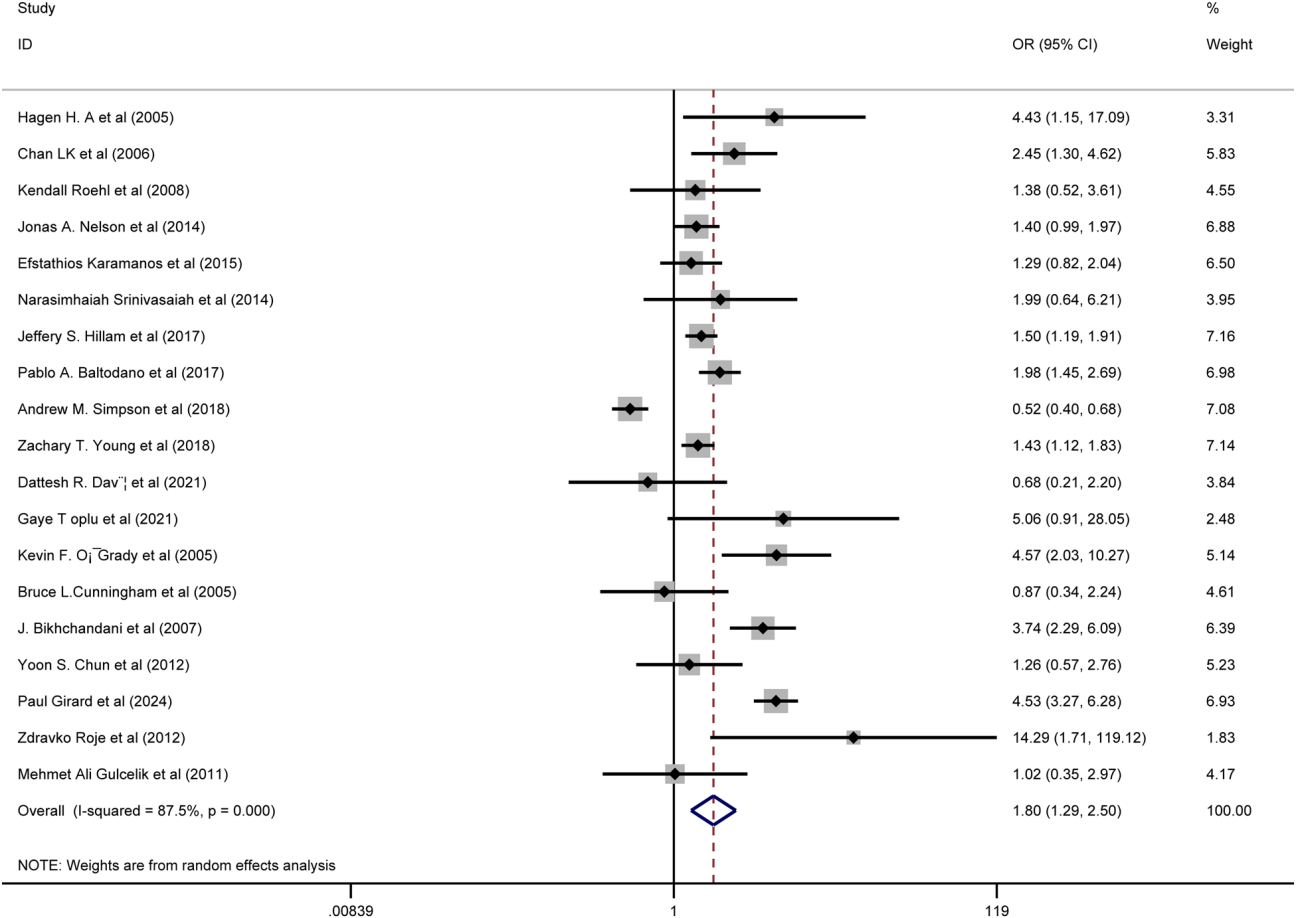
Association between smoking and any complications

Pooled results from nineteen studies showed that smoking was significantly associated with an increased risk of any complications (OR = 1.80, 95% CI 1.29–2.50, p = 0.000; I^2^ = 87.5%) Fig. 3. Subgroup analyses demonstrated a significantly higher incidence of wound dehiscence (OR = 1.84, 95% CI 1.35–2.52, p = 0.000; I^2^ = 36.5%), and wound infection (OR = 1.86, 95% CI 1.57–2.22, p = 0.000; I^2^ = 0.0%) in smokers following RM. No significant associations were found between smoking and hematoma, or medical complications.

#### Associations of surgical techniques with complications

Pooled results from three studies showed that IP was significantly associated with an increased risk of any complications compared to MP (OR = 2.34, 95% CI 1.48–3.72, p = 0.000; I^2^ = 47.0%). Subgroup analyses demonstrated a significantly higher incidence of wound dehiscence (OR = 3.39, 95% CI 1.83–6.29, p = 0.000; I^2^ = 43.5%) in patients who underwent IP. No significant differences were observed between the two surgical techniques in terms of fat necrosis, hematoma, seroma, wound infection, or NAC.

Pooled results from twelve studies showed that IP was significantly associated with an increased risk of any complications compared to SMP (OR = 1.59, 95% CI 1.27–1.99, p = 0.000; I^2^ = 27.5%). Subgroup analyses demonstrated a significantly higher incidence of wound infection (OR = 2.15, 95% CI 1.57–2.94, p = 0.000; I^2^ = 7.8%) and a significantly lower incidence of seroma (OR = 0.28, 95% CI 0.09–0.80, p = 0.018; I^2^ = 0.0%) in patients who underwent IP. No significant differences were observed between the two surgical techniques in terms of fat necrosis, hematoma, nipple sensory loss, skin necrosis, wound dehiscence, hypertrophic scarring, reoperation, or NAC.

No significant differences were observed between IP and SP in terms of any complications, nipple sensory loss, fat necrosis, hematoma, hypertrophic scarring, wound infection, NAC, reoperation, seroma, or wound dehiscence.

Pooled results from three studies showed that SP was significantly associated with an decreased risk of any complications compared to SMP (OR = 0.59, 95% CI 0.37–0.95, p = 0.028; I^2^ = 0.0%). No significant differences were observed between the two surgical techniques in terms of hematoma, wound infection, NAC, or wound dehiscence.

### Sensitivity analysis

We conducted sensitivity analyses for all meta-analyses involving at least five studies by removing each study individually (Supplementary Fig. 1). Only one study affected result stability in analyses of diabetes and any complications, surgical techniques (IP vs SMP) and wound dehiscence, surgical techniques (IP vs SMP) and wound infection, and surgical techniques (IP vs SP) and wound infection. Sensitivity analyses indicated that most results were robust. However, in certain comparisons, the overall effect estimates were sensitive to the exclusion of individual studies, suggesting potential instability. These associations should therefore be interpreted with caution, and further studies are warranted to confirm these findings.

### Publication bias

**Fig. 4:**
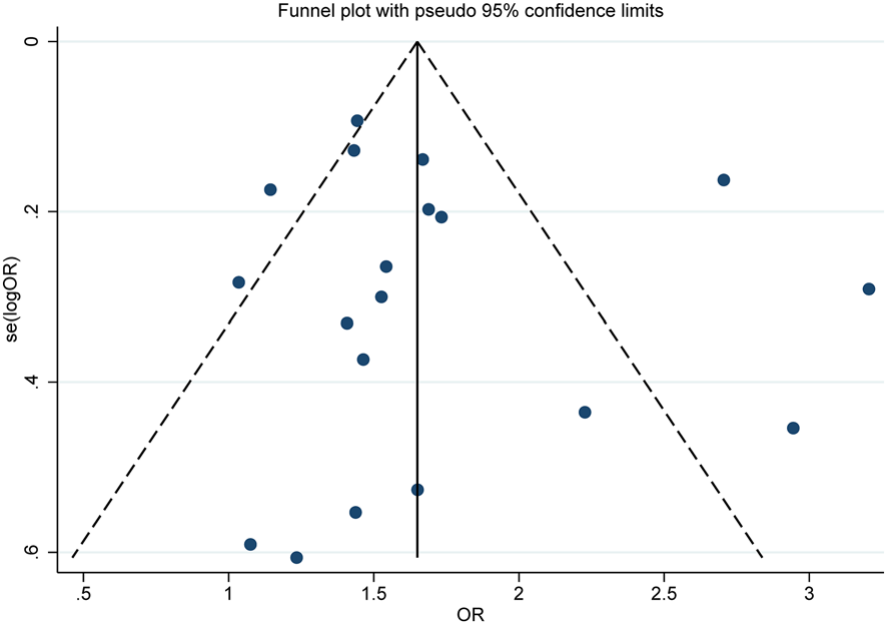
Funnel plot of the association between body mass index ≥ 30 kg/m^2^ and any complications

Publication bias was evaluated using funnel plots and Egger’s test (Fig. 4 and Table 3). According to Egger’s test, evidence of publication bias was found only in the analysis of BMI and wound infection (p = 0.044).

## Discussion

As RM becomes more popular in clinics, postoperative complications have received increasing attention. We conducted meta-analyses of sixty-one studies to identify risk factors for complications following RM, finding that BMI ≥ 30 kg/m^2^, smoking, diabetes, surgical techniques, and PRT were significantly associated with risk of any complications. Furthermore, associations between risk factors and specific complications were also researched. BMI ≥ 30 kg/m^2^ was linked to higher risks of delayed healing, fat necrosis, wound infection, and dehiscence. PRT increased the risks of fat necrosis, infection, and seroma. Smoking was associated with higher risks of wound infection and dehiscence. Compared to MP, IP had a higher risk of dehiscence; compared to SMP, IP had a higher risk of infection but a lower risk of seroma.

BMI is commonly considered a risk factor for postoperative complications [1, 71]. According to the WHO BMI classification, we categorized patients into a non-obesity group (BMI < 30 kg/m^2^) and an obesity group (BMI ≥ 30 kg/m^2^) [25, 72]. Consistent with previous study [40, 57], our results showed that obesity significantly increased postoperative complications, particularly wound complications. The increased postoperative complications may be attributed to more challenging surgical exposure, longer operative time, and a higher incidence of wound fat liquefaction in obese patients. However, most patients with macromastia are obese, which presents challenges for surgeons in both preoperative assessment and postoperative management to prevent wound complications.

Smoking was a significant risk factor for postoperative complications, particularly affecting wound healing. Our study found that smoking increased the risk of wound infection by 1.86-fold and wound dehiscence by 1.84-fold. Nicotine can damage the endothelial wall and reduce the blood's ability to transport oxygen. Nicotine-induced vasoconstriction leads to poor wound perfusion, which delays healing and contributes to wound infection and dehiscence [1, 73]. Moreover, smoking can weaken the immune system's ability to combat infections [74]. Poor wound healing increases patients'economic burden and induces anxiety and negative emotions. Therefore, patients are advised to quit smoking, with a general recommendation to abstain from tobacco use for at least four weeks prior to RM surgery [26].

Our study identified diabetes as a risk factor, increasing the incidence of any complications by 1.41-fold after RM. However, previous studies have reported inconsistent results, possibly due to insufficient data and the absence of subgroup analyses for specific complications [27, 75]. Diabetes is a major factor contributing to delayed wound healing. Normally, macrophages are crucial for tissue repair and the transition from pro-inflammatory to anti-inflammatory phenotypes [76–78]. However, diabetes disrupts macrophage function, impeding monocyte recruitment, reducing phagocytosis, and preventing this transition [79]. Furthermore, diabetes impairs keratinocyte and fibroblast function, hindering wound epithelialization [10]. Therefore, blood glucose levels should be monitored and controlled to optimize perioperative management in diabetic patients and improve postoperative outcomes.

PRT can impair tissue healing after RM, increasing the risk of wound infection [42]. Radiation causes fibrosis, reduced vascularity, and altered immune response, which hinder tissue regeneration and healing. This makes irradiated skin more susceptible to complications. The tension on incision sites in RM, particularly after large tissue resections, can further exacerbate these risks. Careful surgical planning and postoperative management, including minimizing tension on incisions and ensuring proper wound care, are essential to reduce infection risks in patients with a history of PRT.

Various techniques are used in RM; however, due to limited data, only the IP, MP, SMP, and SP techniques were included in our study. Clinically, IP remains one of the most commonly used methods [80]. We found that IP was associated with a higher risk of wound dehiscence compared to MP, likely due to its longer and heavier pedicle, which increases tension at the incision site and may compromise perfusion. In contrast, the MP technique preserves medial blood supply and requires less tissue undermining, thereby promoting better healing and reducing wound stress [64].

Compared to SMP, IP was linked to a higher risk of wound infection but a lower risk of seroma. The increased infection risk may result from the longer pedicle and more extensive dissection involved in IP, especially in large-volume reductions, which can impair perfusion and increase tissue tension at the inferior pole [81]. Additionally, more extensive tissue handling may elevate the risk of contamination. However, IP may disrupt fewer lymphatic channels and preserve more surrounding tissue, promoting better lymphatic drainage and reducing fluid accumulation, thus lowering the risk of seroma. In contrast, SMP involves more extensive mobilization of medial and superior tissues, which may create larger dead spaces and compromise lymphatic flow [82].

Furthermore, our study showed that SP was associated with fewer overall complications than SMP. This could be attributed to SP’s shorter pedicle and limited dissection, which help preserve vascular integrity and reduce tension. A retrospective study supports this finding, reporting a complication rate of 22% for SP versus 36% for SMP [32]. Nonetheless, no single technique suits all patients; individualized surgical planning based on breast anatomy, patient preferences, and healing potential remains essential for optimal outcomes.

This meta-analysis has several limitations. The majority of the included studies were retrospective, which reduces the strength of the evidence supporting our conclusions. The included studies varied in design, sample size, and quality, which may contribute to heterogeneity and affect the overall reliability of the pooled results. Potential publication bias could not be entirely excluded, as studies with negative or inconclusive results are less likely to be published. Some studies lacked complete data on important covariates or outcome measures, limiting our ability to perform more detailed subgroup or sensitivity analyses. Despite using rigorous inclusion criteria and a comprehensive search strategy, the possibility of missing relevant studies remains. Finally, as with all meta-analyses, our findings are inherently dependent on the quality and accuracy of the original studies, and residual confounding may still exist. Future studies should focus on high-quality, prospective, and multicenter designs to validate the associations identified in this meta-analysis. Standardized definitions of complications and surgical techniques are needed to reduce heterogeneity. Moreover, individual patient data meta-analyses could allow for more precise risk stratification and adjustment for confounding factors.

## Conclusions

In conclusion, BMI, smoking, diabetes, PRT, and surgical techniques significantly influence the risk of complications after RM. These findings highlight the importance of comprehensive preoperative risk assessment and individualized surgical planning to minimize postoperative complications and improve patient outcomes.

## Supplementary Information

Supplementary Material 1.

Supplementary Material 2.

## Data Availability

The datasets supporting the conclusions of this article are included within the article and its additional files.

## Abbreviations

RM: Reduction mammaplasty
NAC: Nipple-areola complex necrosis
NOS: Newcastle–Ottawa Scale
BMI: Body mass index
TRW: Tissue resection weight per breast
PRT: Previous radiation therapy
IP: Inferior pedicle
MP: Medial pedicle
SP: Superior pedicle
SMP: Superomedial pedicle
OR: Odds ratio
CI: Confidence interval
