# Supplementary material for: Risk factors for complications after reduction mammaplasty: a systematic review and meta-analysis
Source: Eur J Med Res. 2025 Jun 2;30:440. doi: 10.1186/s40001-025-02723-z (PMC12128374; doi:10.1186/s40001-025-02723-z)
Supplement: Supplementary file 1 — Supplementary Material 1. [file 40001_2025_2723_MOESM1_ESM.docx]

Supplementary Fig. 1 Sensitivity analysis

Age and any complications


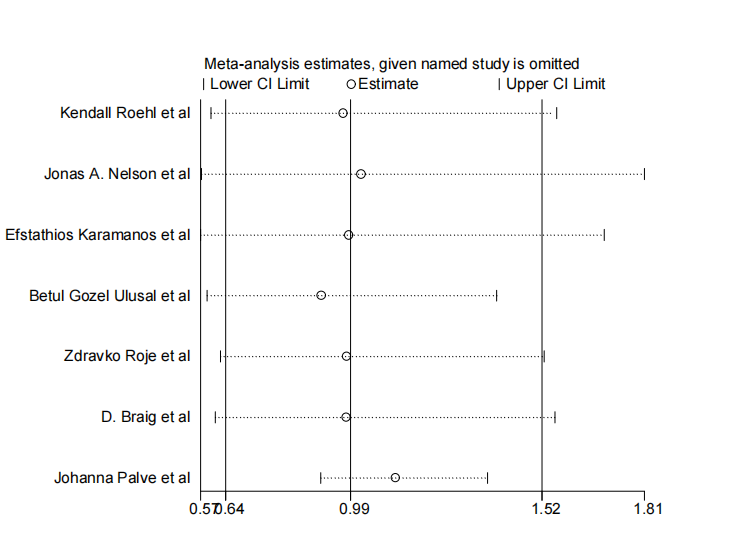


BMI and any complications


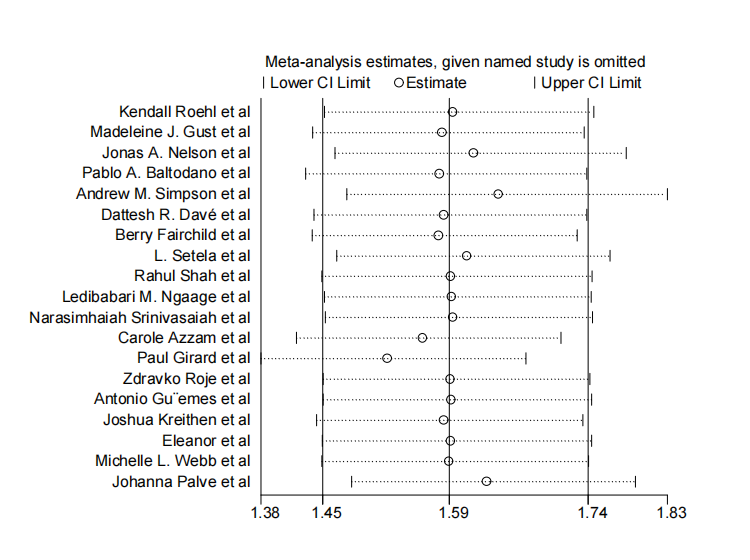


BMI and wound dehiscence


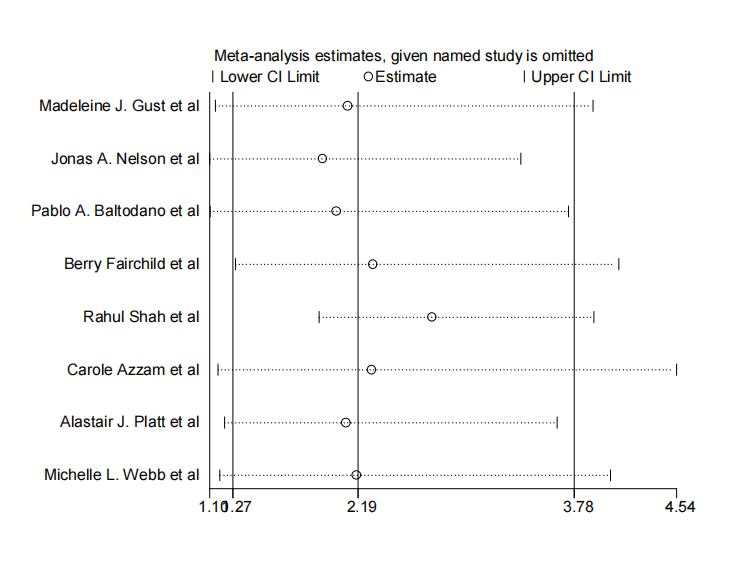


BMI and wound infection


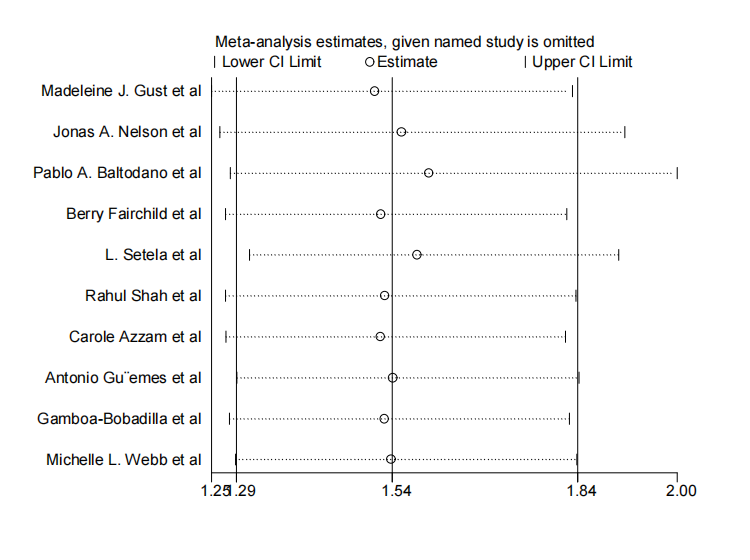


Diabetes and any complications


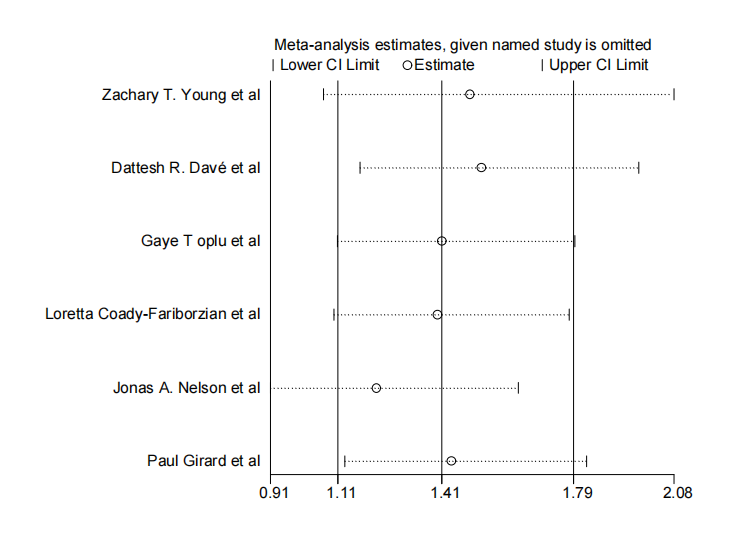


PRT and any complications


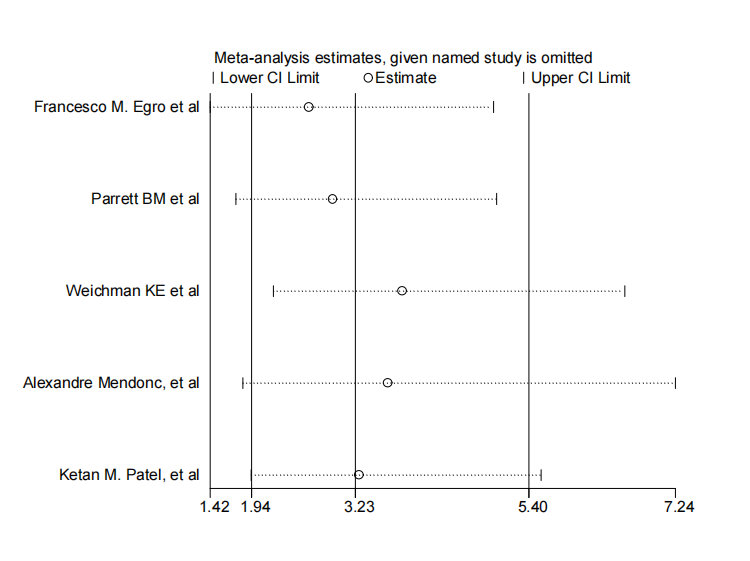


Smoking and any complications


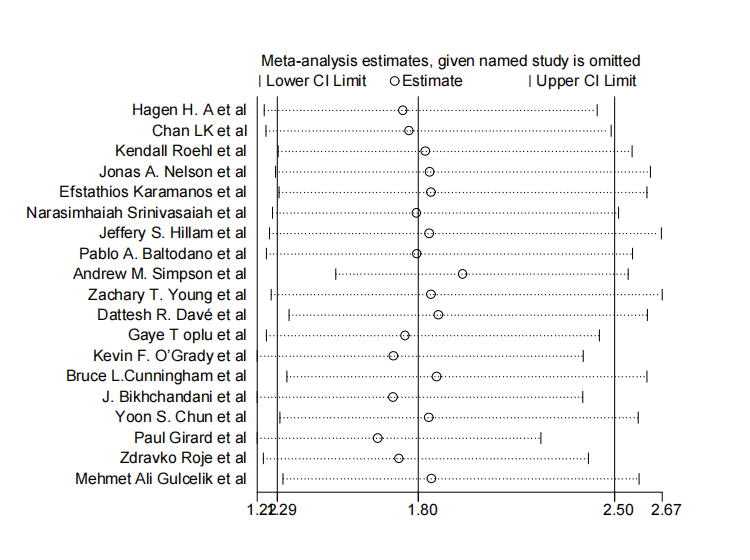


Smoking and hematoma


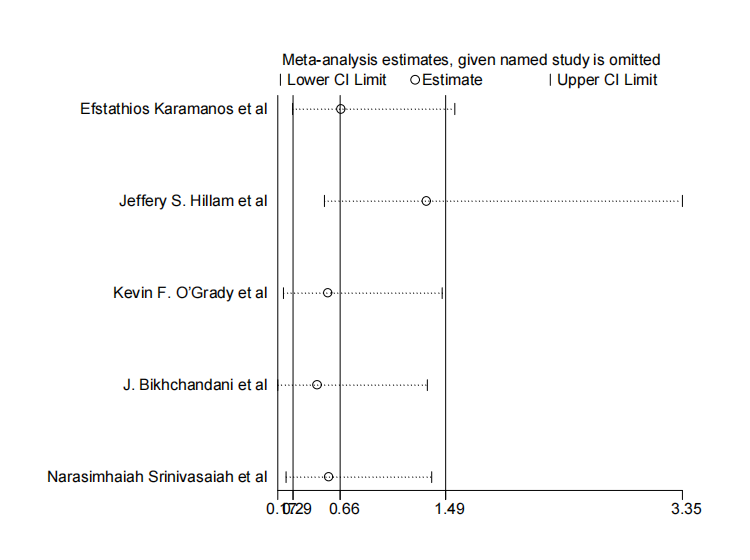


Smoking and wound dehiscence


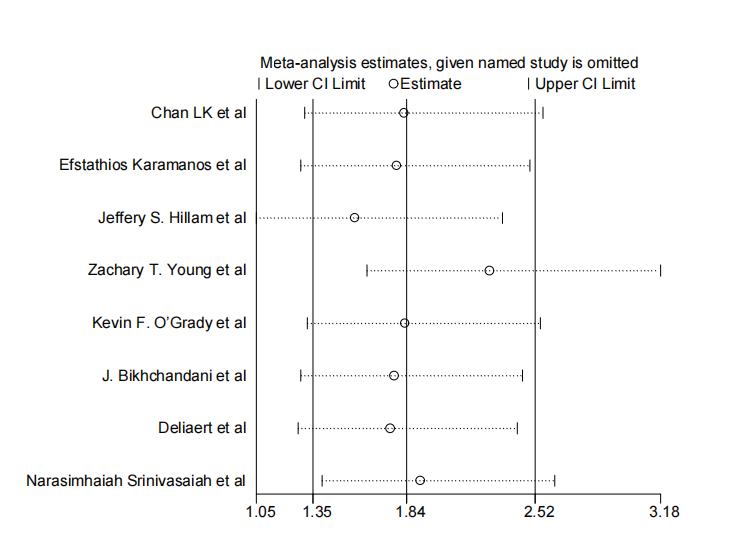


Smoking and wound infection


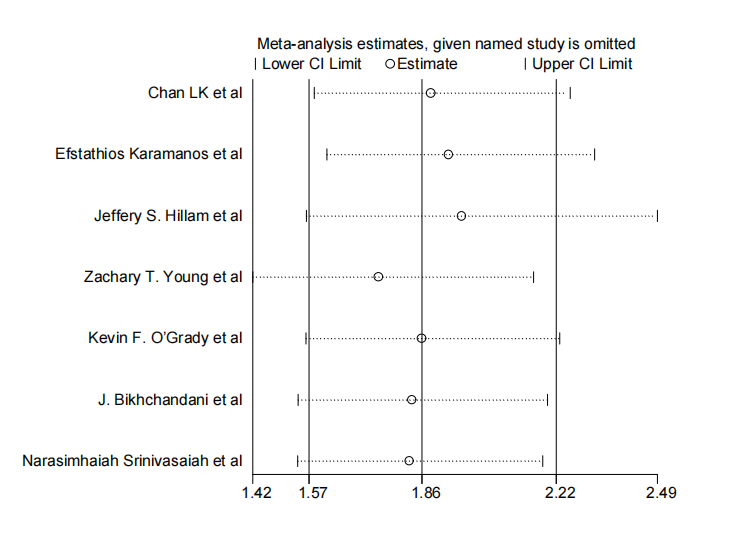


TRW and any complications


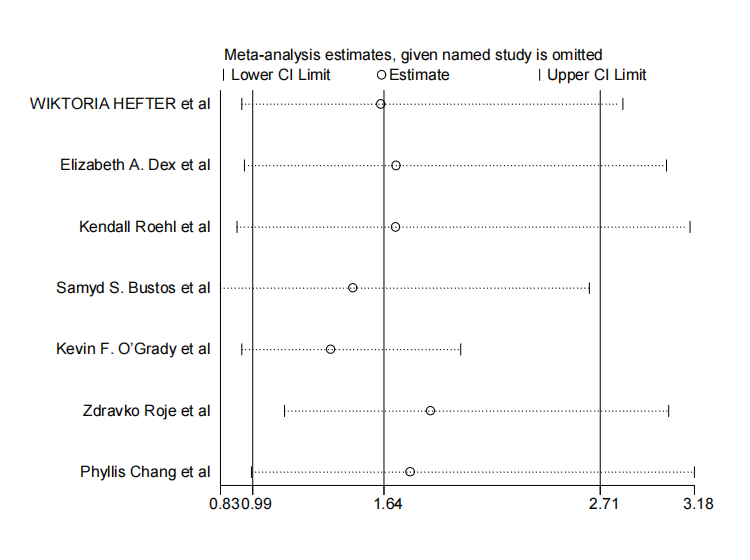


Surgical technique and any complications (IP and SMP)


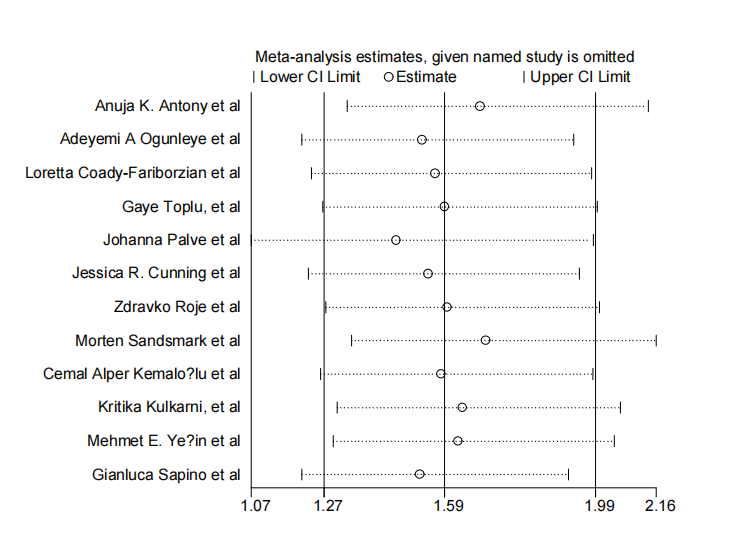


Surgical technique and fat necrosis (IP and SMP)


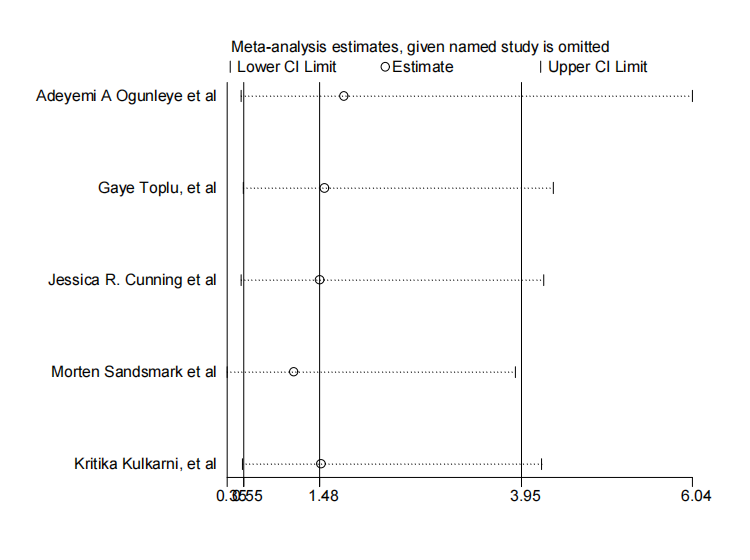


Surgical technique and hematoma (IP and SMP)


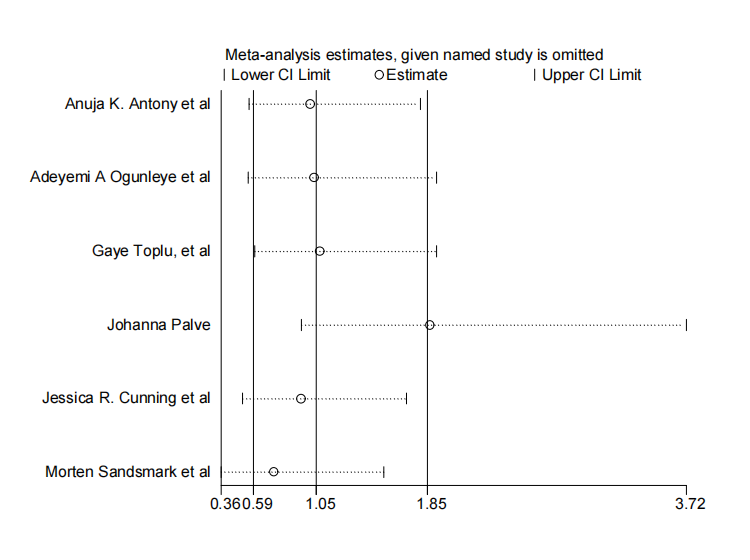


Surgical technique and NAC (IP and SMP)


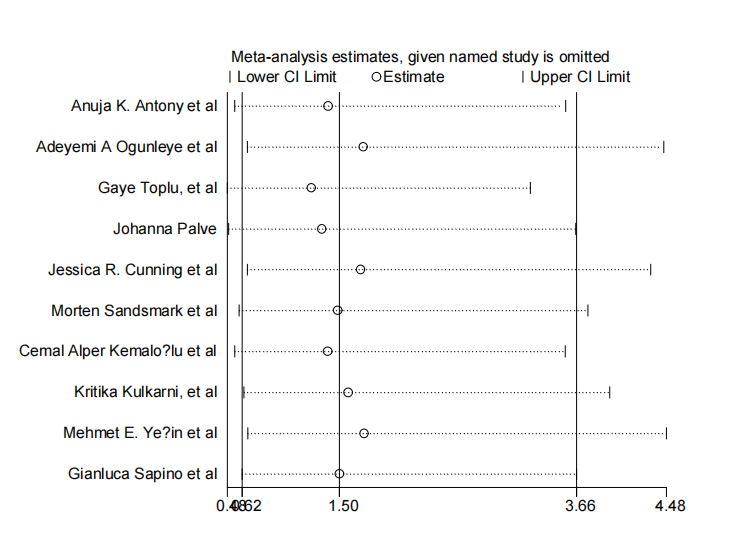


Surgical technique and wound dehiscence (IP and SMP)


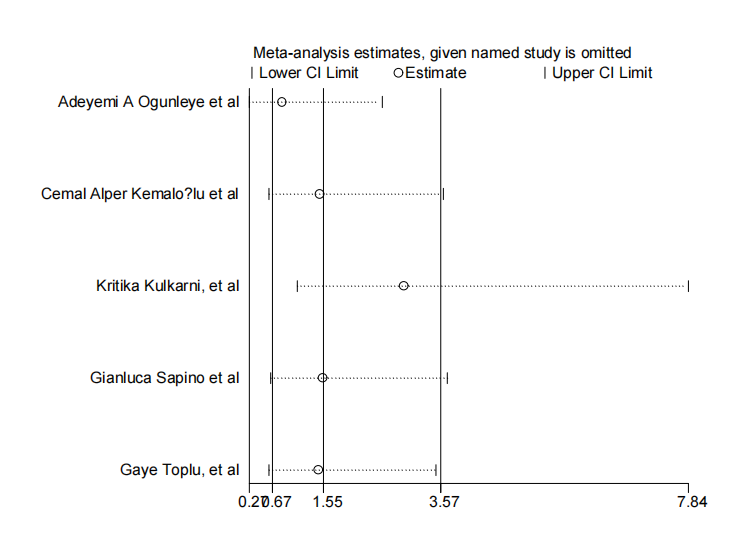


Surgical technique and wound infection (IP and SMP)


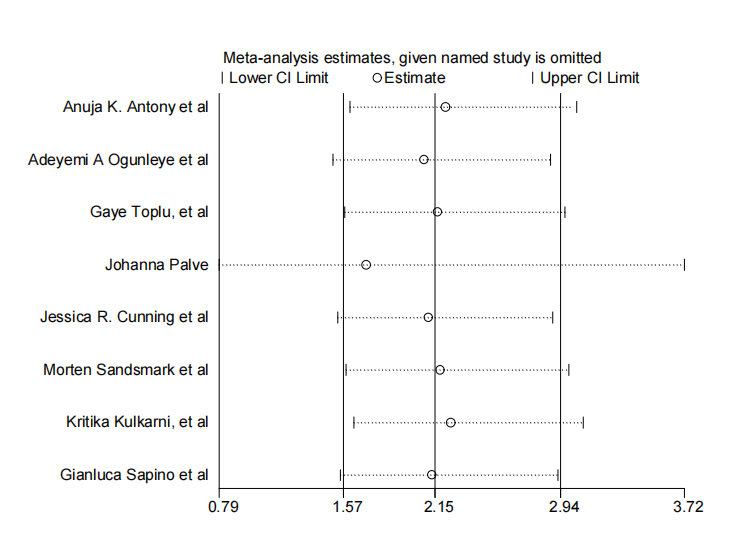


Surgical technique and any complications (IP and SP)


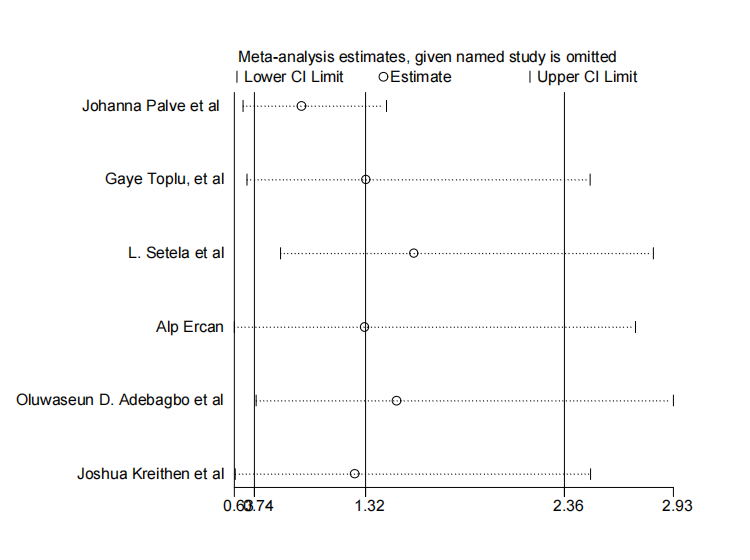


Surgical technique and wound infection (IP and SP)
