# Supplementary material for: Risk factors for complications after reduction mammaplasty: a systematic review and meta-analysis
Source: Eur J Med Res. 2025 Jun 2;30:440. doi: 10.1186/s40001-025-02723-z (PMC12128374; doi:10.1186/s40001-025-02723-z)
Supplement: Supplementary file 2 — Supplementary Material 2. [file 40001_2025_2723_MOESM2_ESM.docx]

List of excluded articles and the reasons for their exclusion.

| Reasons | PMID |
| --- | --- |
| Existing other mixed breast surgical procedure simultaneously | - 19371840, 24324279, 26816558, 28350649, 34640438, 36294707, 36820864, 30805691, 12477471, 18090737 |
| Not case-control, cohort, cross-sectional, or randomized controlled study | 8852780, 16374091, 17580033, 25668497, 31481711, 29948104, 18300952, 19371840, 19083524, 1561266, 9810985, 36534097, 23849259, 10845327, 37153830, |
| Lack necessary data | 23806954, 11039370, 29213346, 22658727, 22258836, 15731673, 15793442, 33561191, 23627557 |
| Letter | **16** 16816721 |
